# Supplementary material for: Elucidating the catalytic mechanism of Prussian blue nanozymes with self-increasing catalytic activity
Source: Nat Commun. 2024 Jul 13;15:5908. doi: 10.1038/s41467-024-50344-7 (PMC11246500; doi:10.1038/s41467-024-50344-7)
Supplement: Supplementary file 1 — Supplementary Information [file 41467_2024_50344_MOESM1_ESM.pdf]

Supplementary Information

Elucidating the catalytic mechanism of Prussian blue nanozymes with self-increasing catalytic activity

Kaizheng Feng[1], Zhenzhen Wang[2], Shi Wang[1], Guancheng Wang[1], Haijiao Dong[3], Hongliang He[1], Haoan Wu[1], Ming Ma[1*], Xingfa Gao[2*] and Yu Zhang[1*]

[1]State Key Laboratory of Digital Medical Engineering, Jiangsu Key Laboratory for Biomaterials and Devices, School of Biological Science and Medical Engineering & Basic Medicine Research and Innovation Center of Ministry of Education, Zhongda Hospital, Southeast University, Nanjing 211102, China. [2]Laboratory of Theoretical and Computational Nanoscience, National Center for Nanoscience and Technology of China, Beijing 100190, China. [3]Nanjing Institute of Measurement and Testing Technology, Nanjing 210049, China.

*Corresponding author

Email: maming@seu.edu.cn, gaoxf@nanoctr.cn, zhangyu@seu.edu.cn

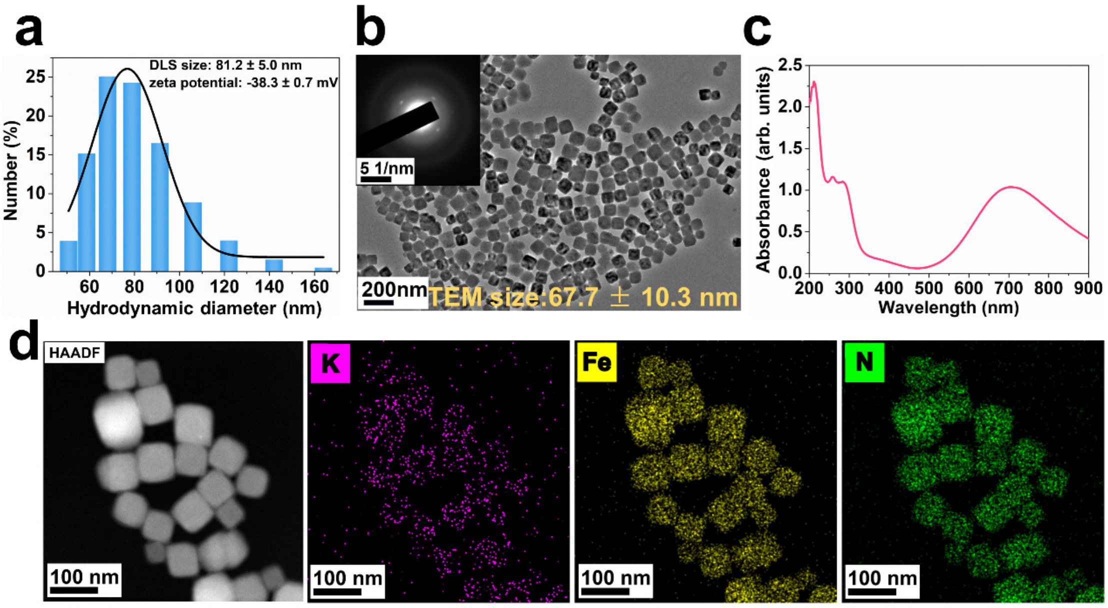

Supplementary Fig. 1 Synthesis and characterization of 81 nm PBNZ. a DLS measurement. **b** TEM and electron diffraction images. **c** UV-vis spectrum. **d** Element mapping images. Images were collected three times with similar results.

Supplementary discussion for Supplementary Fig. 1: PBNZ with a hydrodynamic diameter of 81.2 ± 5.0 nm were prepared when x = 1.0 mL with a shortened reaction time of 25 min. The zeta potential of 81 nm PBNZ was -38.3 ± 0.7 mV (Supplementary Fig. 1a). The TEM and electronic diffraction images showed their cubic morphology and low crystallinity (Supplementary Fig. 1b). The maximum UV-vis absorption peak of 81 nm PBNZ was located near the wavelength of 700 nm due to the inter-metal charge transfer from C-coordinated Fe (II) to N-coordinated Fe (III) (Supplementary Fig. 1c). Additionally, the element mapping analysis demonstrated the existence of K, Fe and N elements in the particles (Supplementary Fig. 1d).

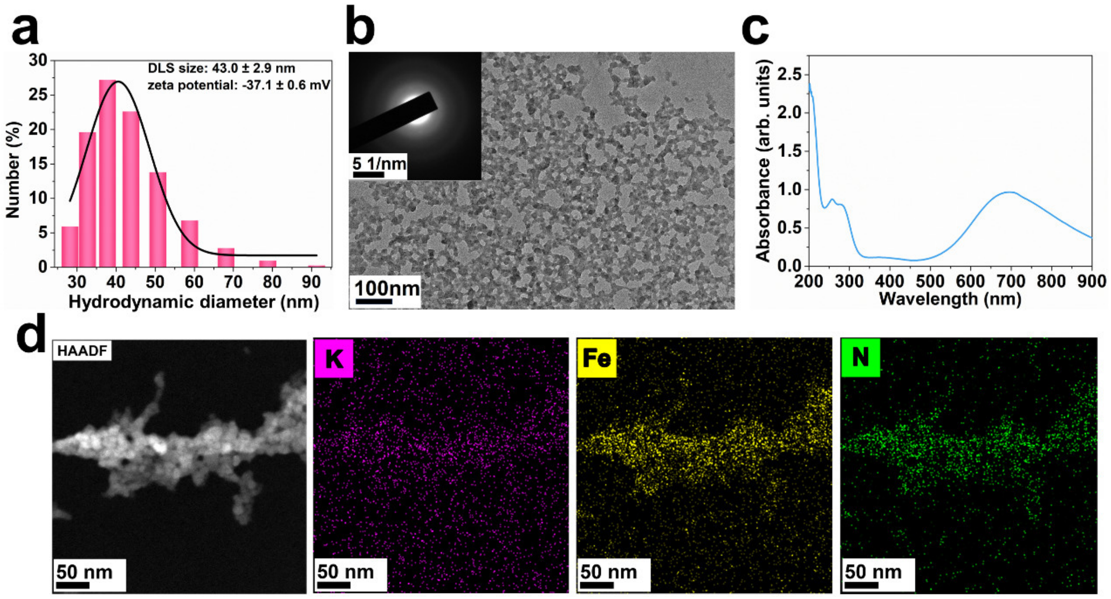

Supplementary Fig. 2 Synthesis and characterization of 43 nm PBNZ. a DLS measurement. **b** TEM and electron diffraction images. **c** UV-vis spectrum. **d** Element mapping images. Images were collected three times with similar results.

Supplementary discussion for Supplementary Fig. 2: As the *x* value increased to 20.0 mL, PBNZ with a hydrodynamic diameter of 43 ± 2.9 nm and zeta potential of -37.1 ± 0.6 mV were obtained (Supplementary Fig. 2a). The TEM and electronic diffraction images showed their irregular morphology and low crystallinity (Supplementary Fig. 2b). Notably, the aggregation of the PBNZ observed in the TEM images was due to the drying process during the TEM sample preparation (the particle size is thus hard to measure) and will not affect their catalytic behavior in solution[1]. The maximum UV-vis absorption peak of 43 nm PBNZ was also located near the wavelength of 700 nm (Supplementary Fig. 2c). The element mapping analysis revealed the existence of K, Fe and N elements in the particles (Supplementary Fig. 2d).

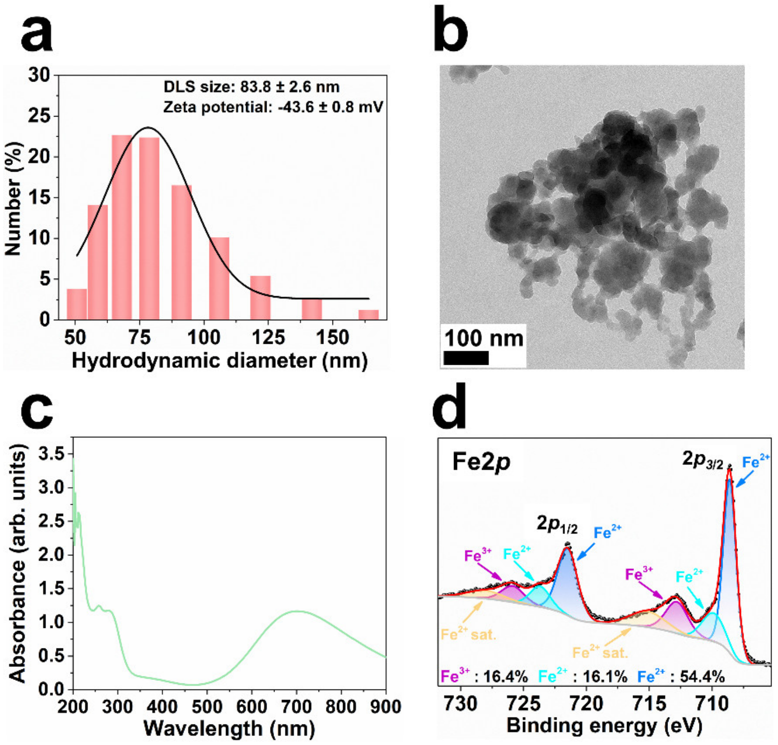

Supplementary Fig. 3 Synthesis and characterization of the PBNZ prepared by Fe^{2+} and $[Fe(CN)_6]^{3-}$. a DLS measurement. **b** TEM image. TEM image was collected three times with similar results. **c** UV-vis spectrum. **d** Fitting XPS spectrum of Fe2*p*.

Supplementary discussion for Supplementary Fig. 3: Fe^{3+} and $[Fe(CN)_6]^{4-}$ were replaced by Fe^{2+} and $[Fe(CN)_6]^{3-}$ to synthesize the irregular PBNZ with a hydrodynamic diameter of 83.8 ± 2.6 nm and a zeta potential of -43.6 ± 0.8 mV (Supplementary Fig. 3a, b). The maximum UV-vis absorption peak of the prepared PBNZ was still located around 700 nm (Supplementary Fig. 3c). Notably, a similar Fe 2*p* spectrum was observed compared with that of the 81 nm and 43 nm PBNZ (Supplementary Fig. 3d).

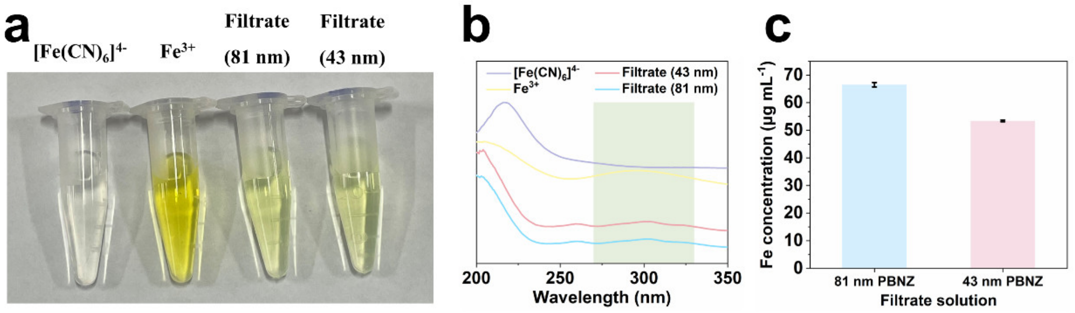

Supplementary Fig. 4 Composition detection on the filtrate solution of unpurified PBNZ. **a** Digital photographs and **b** UV-vis spectra of $[Fe(CN)_6]^{4-}$, Fe^{3+} and filtrate solutions obtained from unpurified PBNZ. **c** Fe element detection on the filtrate solutions by ICP-MS (n = 1 experiment, error bars represent the detective SD of ICP-MS device).

Supplementary discussion for Supplementary Fig. 4: The unpurified PBNZ solution was ultrafiltrated once the synthesis was finished and the color of the obtained filtrates was visibly similar to Fe^{3+} in yellow (Supplementary Fig. 4a). Moreover, a slight adsorption of the filtrate solutions around 300 nm was observed which further verified that the main component in the filtrate solutions was Fe^{3+}, leading to the deficiency of Fe^{3+} in the actual structure of prepared PBNZ (Supplementary Fig. 4b). The concentration of the unreacted Fe^{3+} during the synthesis process was determined by ICP-MS, accounting for 6.5% and 4.8% of the total injected Fe^{3+} for the preparation of 81 nm and 43 nm PBNZ, respectively.

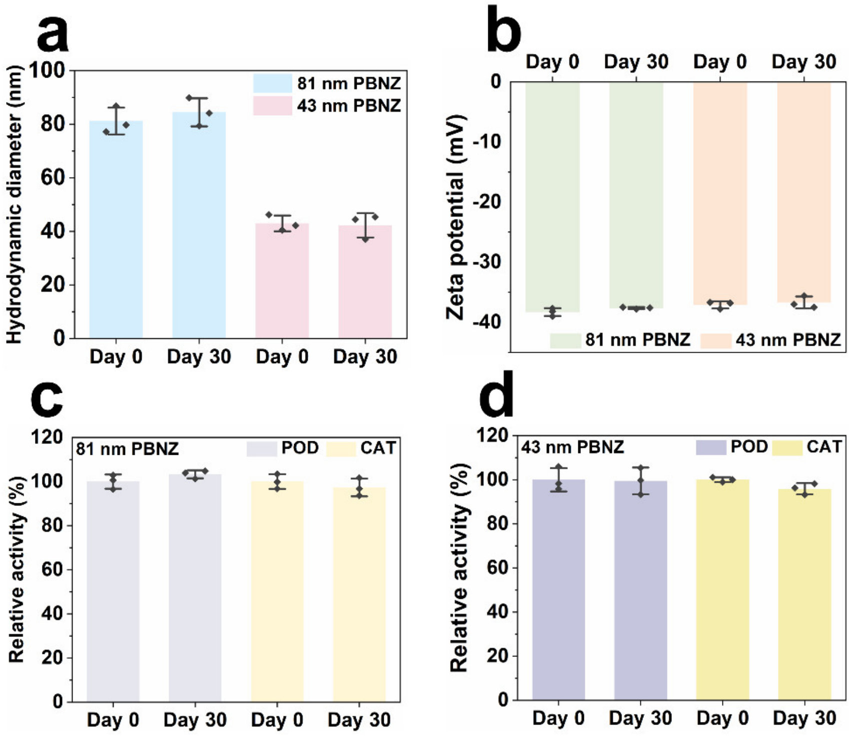

Supplementary Fig. 5 Stability of PBNZ stored in deionized water for 30 days. a Hydrodynamic diameter (n = 3 independent experiments). **b** Zeta potential (n = 3 independent experiments). Relative catalytic activity of **c** 81 nm and **d** 43 nm PBNZ (n = 3 independent experiments). Error bars represent SD from three independent measurements and all data are presented as mean values ± SD.

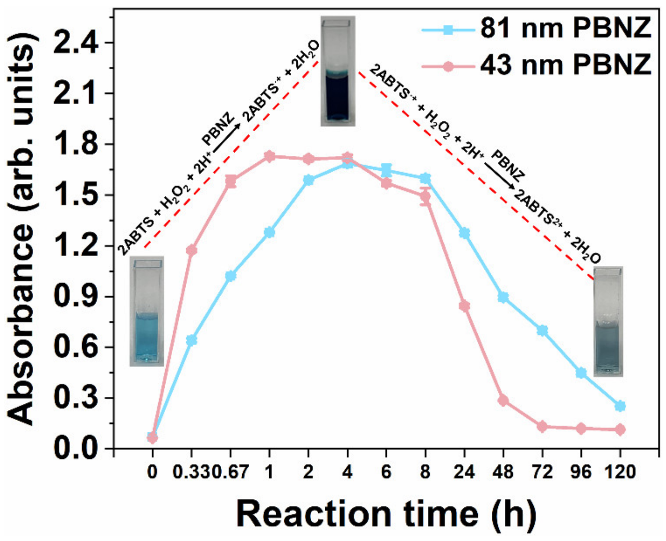

Supplementary Fig. 6 Consecutive oxidation of ABTS catalyzed by PBNZ for 120 h. n = 3 independent experiments, error bars represent SD from three independent measurements and data are presented as mean values ± SD. Inset: digital photographs of the catalytic reaction solution.

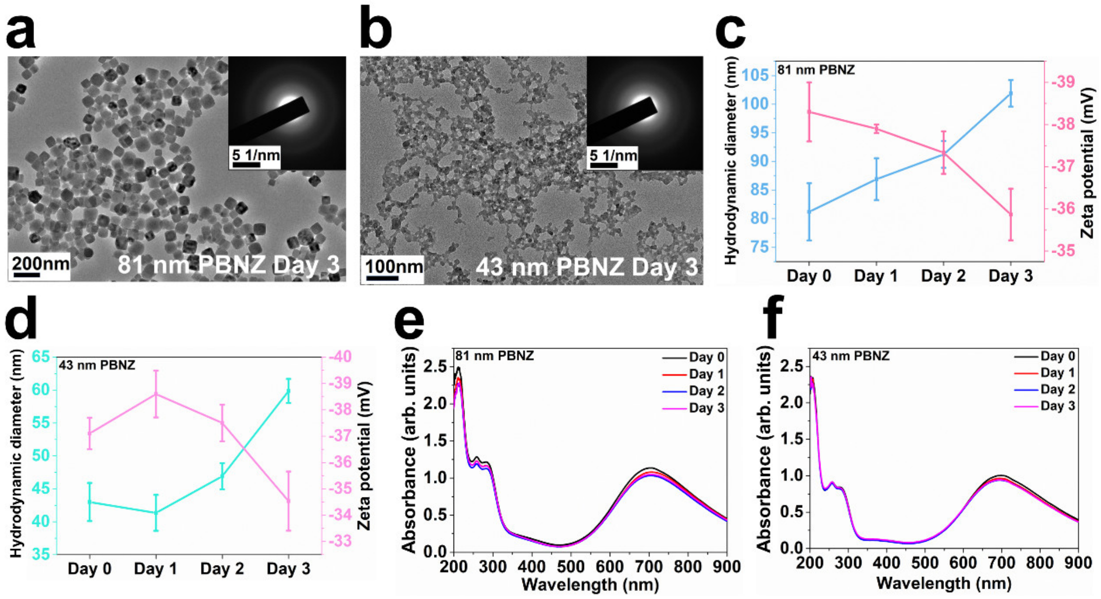

Supplementary Fig. 7 Supporting characterization of the PBNZ recycled from POD-like cyclic catalysis. TEM and electron diffraction images of the recycled **a** 81 nm and **b** 43 nm PBNZ. Images were collected three times with similar results. DLS measurement of the recycled **c** 81 nm and **d** 43 nm PBNZ (n = 3 independent experiments, error bars represent SD from three independent measurements and all data are presented as mean values ± SD). UV-vis spectra of the recycled **e** 81 nm and **f** 43 nm PBNZ.

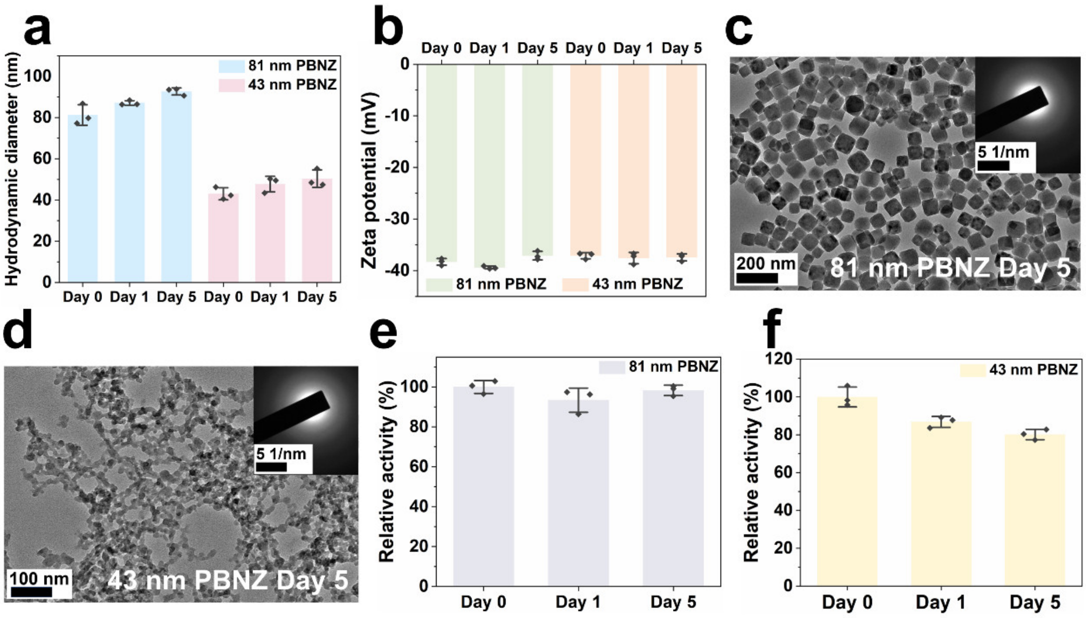

Supplementary Fig. 8 Stability of PBNZ incubated in 0.2 M pH = 3.6 HAc-NaAc buffer. **a** Hydrodynamic diameter (n = 3 independent experiments), **b** zeta potential (n = 3 independent experiments), **c, d** TEM and electron diffraction images, **e, f** POD-like relative activity of the recycled PBNZ (n = 3 independent experiments). Images were collected three times with similar results. Error bars represent SD from three independent measurements and all data are presented as mean values ± SD.

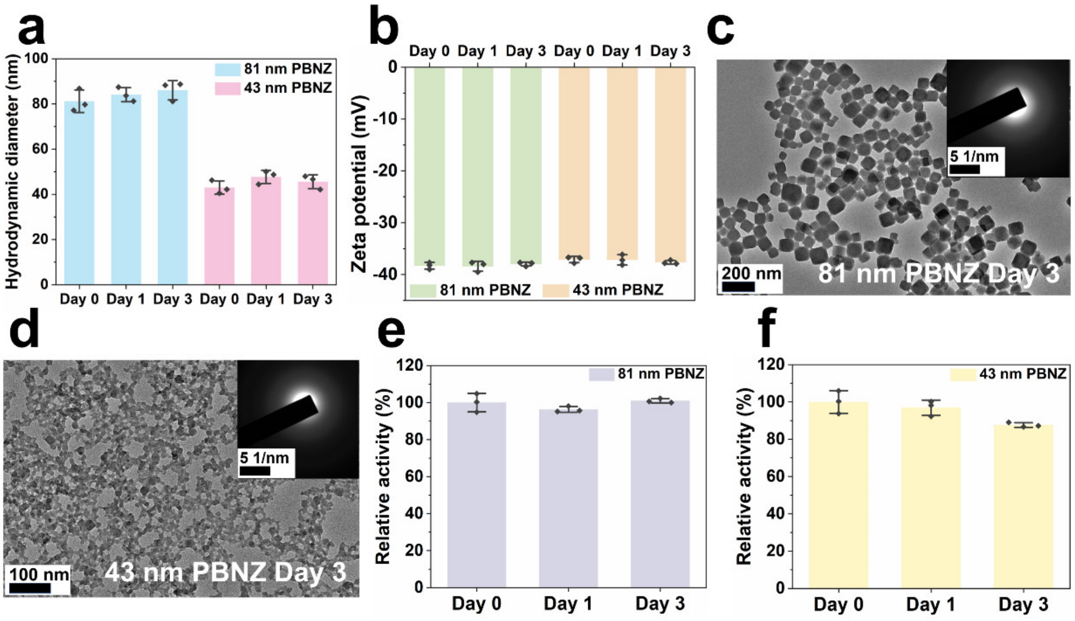

Supplementary Fig. 9 Stability of PBNZ incubated in 0.2 M pH = 3.6 HAc-NaAc buffer containing 0.385 mg mL^{-1} ABTS. a Hydrodynamic diameter (n = 3 independent experiments), **b** zeta potential (n = 3 independent experiments), **c, d** TEM and electron diffraction images, **e, f** POD-like relative activity of the recycled PBNZ (n = 3 independent experiments). Images were collected three times with similar results. Error bars represent SD from three independent measurements and all data are presented as mean values ± SD.

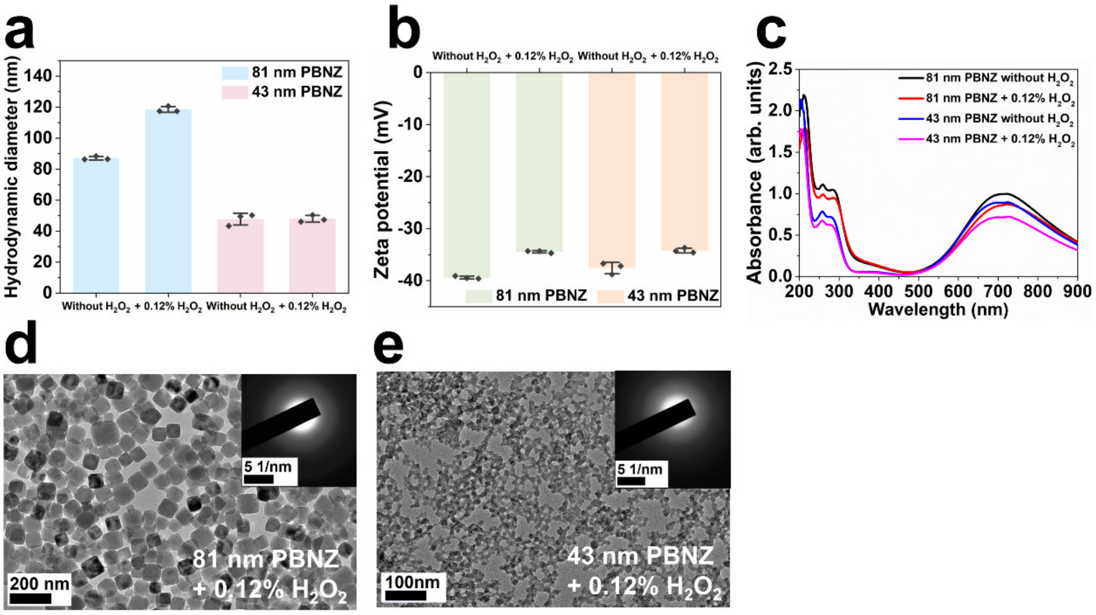

Supplementary Fig. 10 Stability of PBNZ incubated in 0.2 M pH = 3.6 HAc-NaAc buffer containing 0.12% H_2O_2. a Hydrodynamic diameter (n = 3 independent experiments), **b** zeta potential (n = 3 independent experiments), **c** UV-vis spectra, **d, e** TEM and electron diffraction images of the recycled PBNZ. Images were collected three times with similar results. Error bars represent SD from three independent measurements and all data are presented as mean values ± SD.

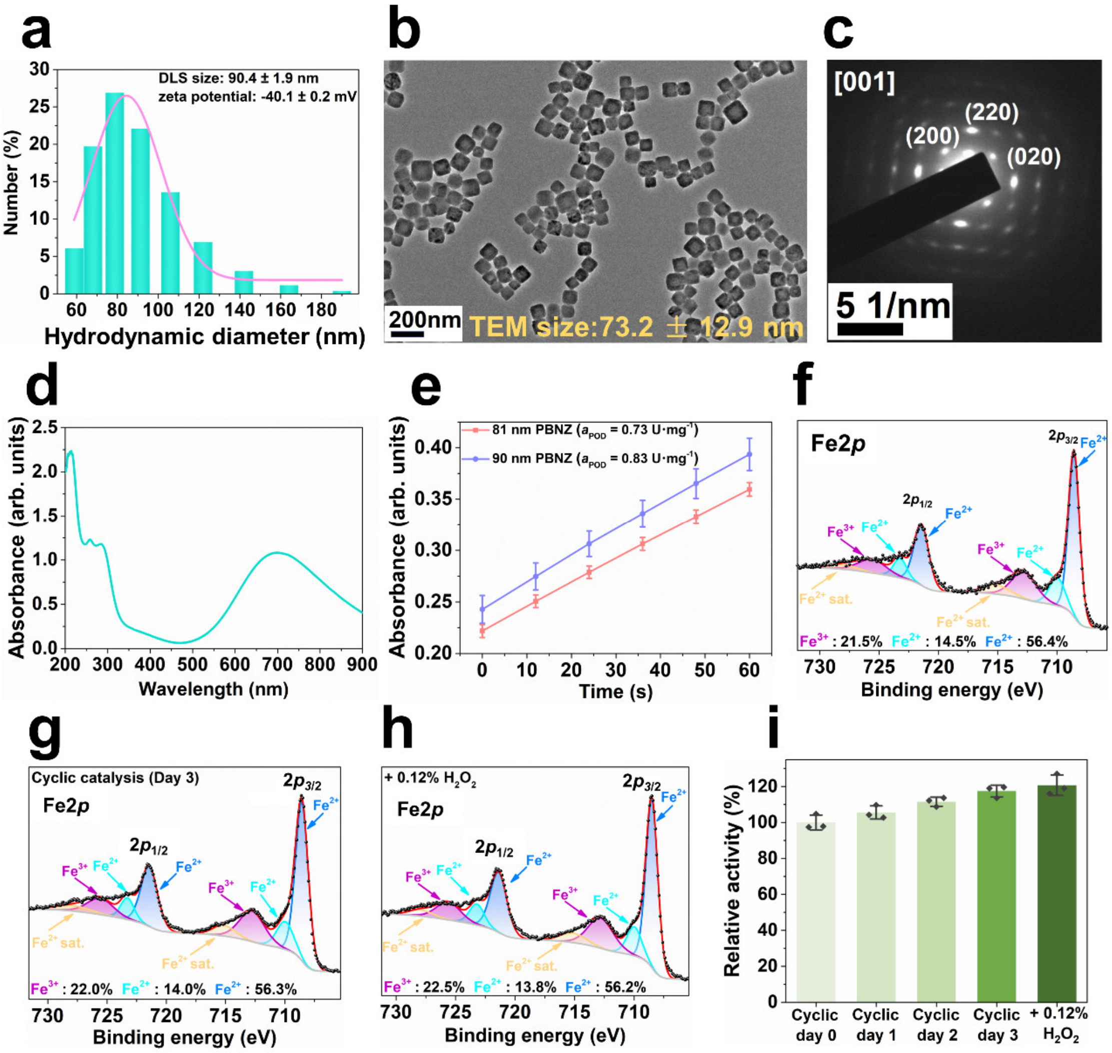

Supplementary Fig. 11 Synthesis and characterization of 90 nm PBNZ. a DLS measurement. **b** TEM image. **c** Electron diffraction image. **d** UV-vis spectrum. **e** POD-like activity (n = 3 independent experiments). Fitting XPS Fe2*p* spectrum of **f** the original PBNZ and the recycled PBNZ from **g** cyclic catalysis or **h** incubation with 0.12% H_2O_2. **i** Relative POD-like activity (n = 3 independent experiments). Images were collected three times with similar results. Error bars represent SD from three independent measurements and all data are presented as mean values ± SD.

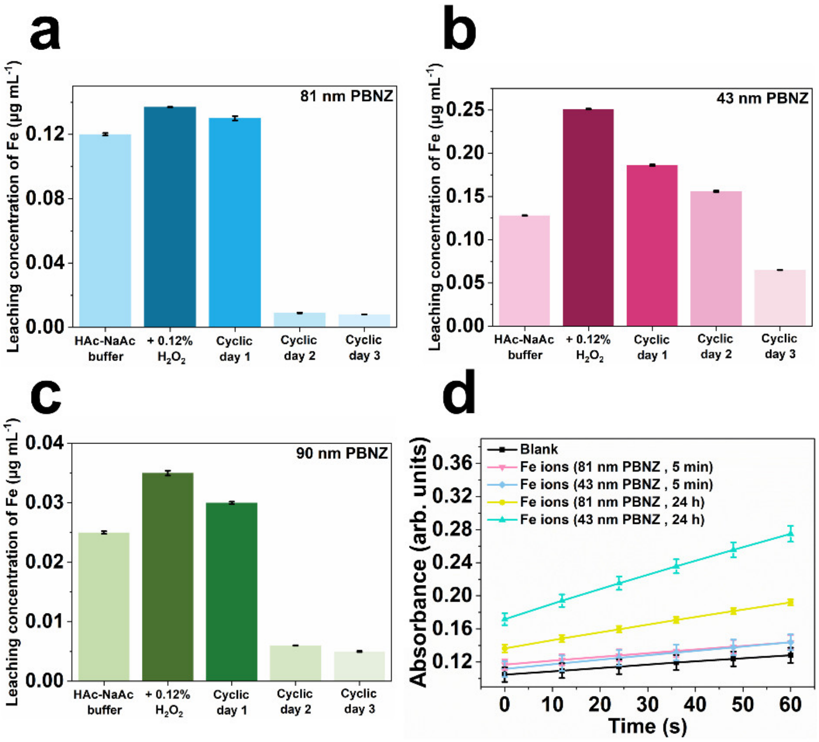

Supplementary Fig. 12 Fe leaching effect on the POD-like activity of PBNZ. Concentration of Fe element in the leaching solution of **a** 81 nm, **b** 43 nm, and **c** 90 nm PBNZ catalytic system (n = 1 experiment, error bars represent the detective standard deviation of ICP-MS device). **d** POD-like activity of leaching solution obtained from the incubation of PBNZ with H_2O_2 (n = 3 independent experiments, error bars represent SD from three independent measurements and data are presented as mean values ± SD).

Supplementary discussion for Supplementary Fig. 12: The leaching solution of PBNZ catalytic system was obtained by ultrafiltration. The concentration of leaching Fe element was measured by ICP-MS. An obvious increase of Fe content was observed after the incubation of PBNZ with 0.12% H_2O_2 or the first round of cyclic catalysis, while the increment after the first round of cyclic catalysis was relatively lower

(Supplementary Fig. 12a-c). Therefore, Fe leaching can reflect the oxidation of PBNZ from another perspective: a higher amount of Fe leaching represents a higher oxidation degree. Therefore, the higher degree of Fe leaching found in the lowly crystalline PBNZ system than that in highly crystalline 90 nm PBNZ reflected the ease of oxidation for PBNZ with low crystallinity. Notably, excessive Fe leaching would cause the loss of active sites on the surface of PBNZ, which detrimentally reduced the increment of their catalytic activity. As a result, although stronger oxidation occurred in 43 nm PBNZ due to their larger specific surface area, the POD-like activity increment of 43 nm PBNZ was not obviously larger than 81 nm PBNZ (Fig. 2b, c).

Since the Fe leaching exists in the long-term catalysis of PBNZ, it is vital to evaluate the catalytic contribution of the leaching Fe ions. The POD-like catalytic ability of the leaching Fe ions obtained from the long-term catalysis was estimated through the following steps: 4.6 μg mL^{-1} PBNZ were incubated with 0.12% H_2O_2 in HAc-NaAc buffer for 24 h. The POD-like activity of the obtained leaching solutions was measured by a direct addition of ABTS and H_2O_2. As seen in Supplementary Fig. 12d, the leaching solution showed a faster absorbance change compared with the control group. Thus, both of the PBNZ and leaching Fe ions contributed for the catalytic activity during the prolonged catalysis. Notably, the catalytic activity of the leaching solution would be exaggerated by the current measurement method, as the ABTS was not added during the incubation and the residual H_2O_2 in the solution was not removed before the measurement. Moreover, apparently reduced Fe leaching was observed when the recycled PBNZ were redispersed into the buffer and experienced the new round of

cyclic catalysis (Supplementary Fig. 12a-c). This means that the catalytic contribution of the leaching solution in prolonged catalysis will continuously decrease as the round of cyclic catalysis increases, ensuring that the catalytic process mostly proceed on the PBNZ nanoparticles in the subsequent cyclic catalysis.

Nevertheless, the main idea for the design of long-term catalysis is to judge the catalytic activity variation of the recycled PBNZ, which was commonly measured in only one minutes. To evaluate the catalytic contribution of leaching Fe in short-term catalytic process, leaching solutions were obtained by ultrafiltration after the incubation of PBNZ with H_2O_2 for 5 minutes. As shown in Supplementary Fig. 12d, the catalytic activity of the leaching solution obtained from 5 minutes incubation was very weak. Therefore, although the gradual Fe leaching during the long-term catalysis competes with the nanoparticles and partially contribute for the catalytic activity, such homogeneous catalysis effect can be ignored during the short-term catalysis. In other words, the short-term catalysis happens on the PBNZ nanoparticles, which will not interfere their catalytic activity measurement and the following mechanism study.

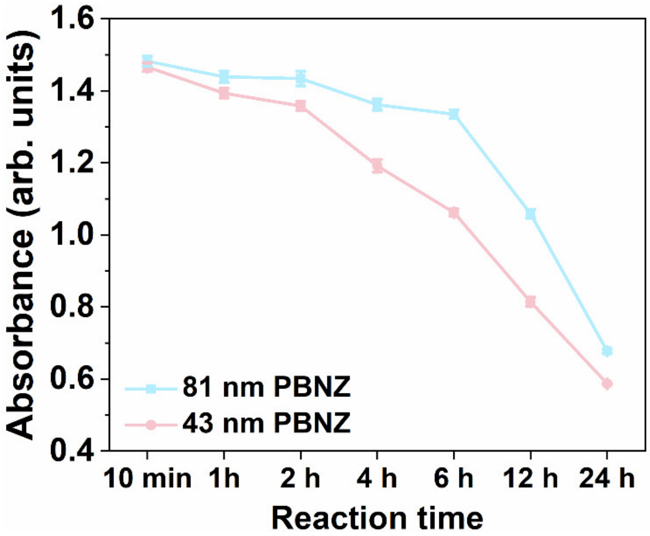

Supplementary Fig. 13 Detection on the self-decomposition process of H_2O_2 catalyzed by PBNZ in 24 h. n = 3 independent experiments, error bars represent SD from three independent measurements and data are presented as mean values ± SD.

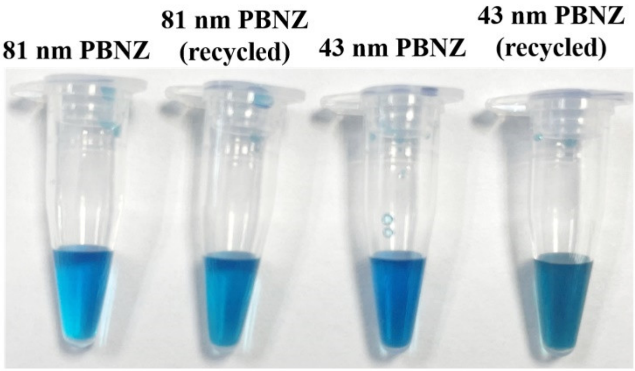

Supplementary Fig. 14 Digital photographs of PBNZ before and after the prolonged CAT-like catalysis.

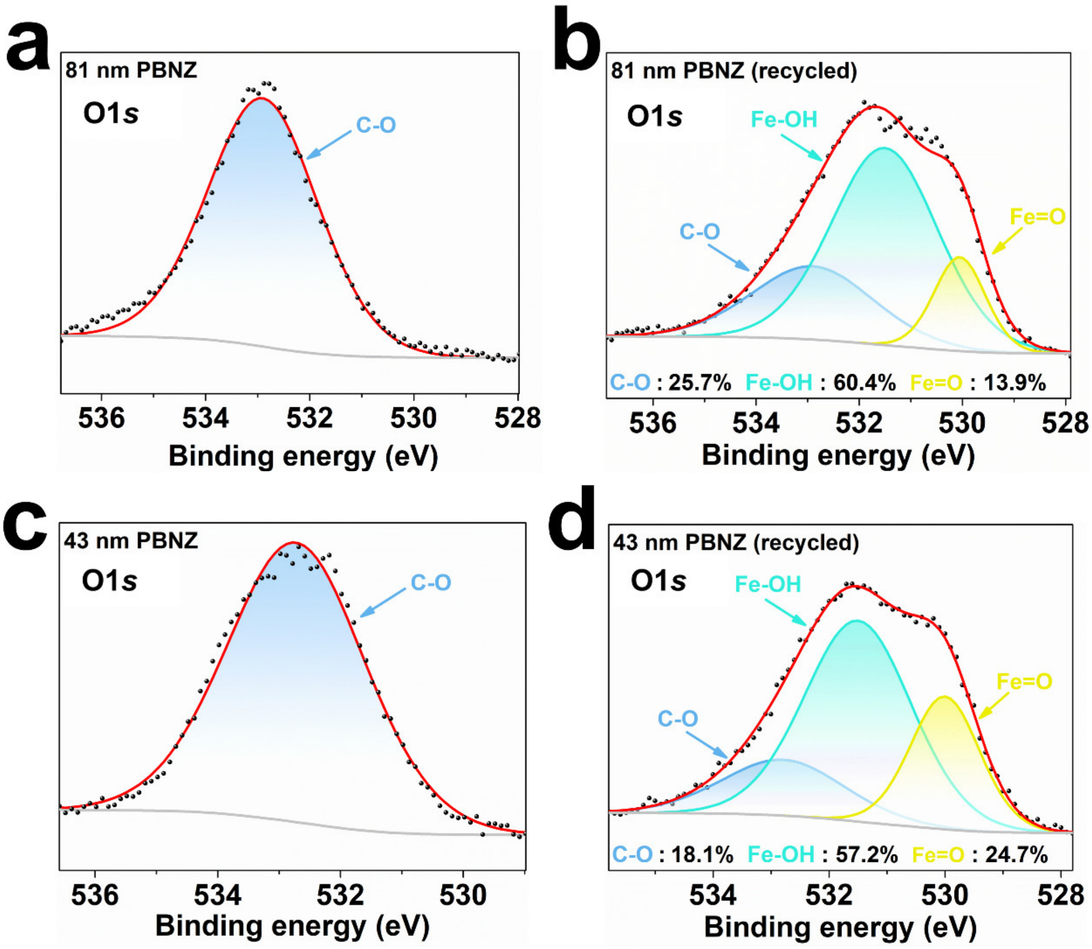

Supplementary Fig. 15 Fitting XPS O1*s* spectrum of PBNZ. O1*s* spectrum of 81 nm PBNZ **a** before and **b** after the prolonged CAT-like catalysis. O1*s* spectrum of 43 nm PBNZ **c** before and **d** after the prolonged CAT-like catalysis.

Supplementary discussion for Supplementary Fig. 15: The spectrum peak of the original PBNZ at 523.9 eV was assigned to the C-O structure originated from the modified citric acid on their surface. While the O1*s* spectra of the recycled PBNZ could be deconvoluted into three peaks corresponding to C-O at 523.9 eV, Fe-OH at 531.5 eV and Fe=O at 530.1 eV[2].

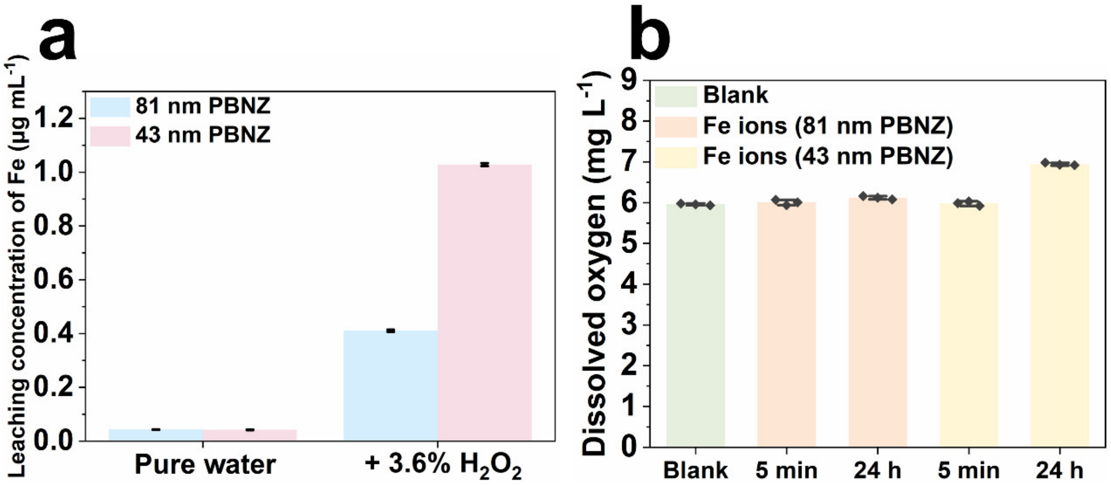

Supplementary Fig. 16 Fe leaching effect on the CAT-like activity of PBNZ. a Concentration of Fe element in leaching solution (n = 1 experiment, error bars represent the detective standard deviation of ICP-MS device). **b** CAT-like activity of leaching solution obtained from the incubation of PBNZ with H_2O_2. Error bars represent the standard deviation from three independent measurements (n = 3 independent experiments, error bars represent SD from three independent measurements and data are presented as mean values ± SD).

Supplementary discussion for Supplementary Fig. 16: An obvious increase of Fe content in leaching solution was observed when PBNZ were incubated with 3.6% H_2O_2 in pure water (Supplementary Fig. 16a). Similar to the situation in POD-like catalysis (Supplementary Fig. 12), the leaching Fe ions contributed for a certain amount of CAT-like activity in long-term catalysis (24 hours) but will not interfere the CAT-like activity measurement of PBNZ in short term (5 minutes) for the following mechanism study.

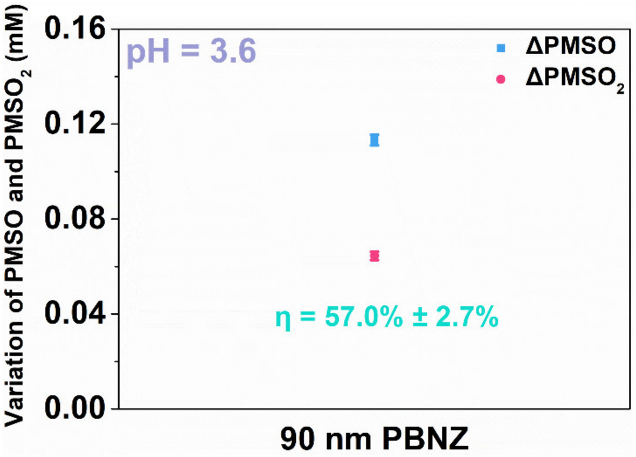

Supplementary Fig. 17 PMSO and $PMSO_2$ detection by HPLC in 90 nm PBNZ system. n = 3 independent experiments, error bars represent SD from three independent measurements and data are presented as mean values ± SD.

Supplementary discussion for Supplementary Fig. 17: As discussed in the previous section, 90 nm PBNZ were harder to be oxidized compared with 81 nm and 43 nm PBNZ. In other words, high valent Fe was more difficult to be generated through the oxidation of crystalline 90 nm PBNZ by H_2O_2, causing the lower transformation efficiency of PMSO/$PMSO_2$ in the detective system of 90 nm PBNZ.

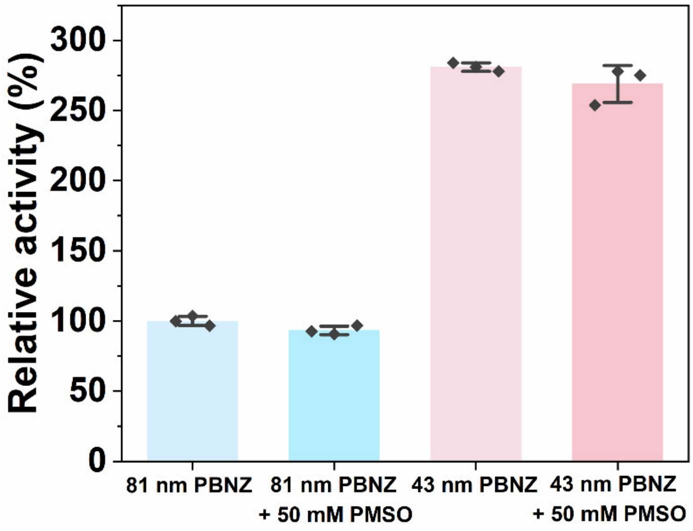

Supplementary Fig. 18 Competitive consumption of the Fe=O by CAT-like

catalysis and oxidation of PMSO. n = 3 independent experiments, error bars represent SD from three independent measurements and data are presented as mean values ± SD.

Supplementary discussion for Supplementary Fig. 18: Only a slight decrease of the CAT-like activity of PBNZ was observed after adding excessive PMSO. In other words, the generated Fe=O would be consumed by the reduction of H_2O_2 to form O_2 under neutral pH, leading to the decreased generation of $PMSO_2$. As a result, an increasing portion of PMSO was oxidized by Fe-OH rather than Fe=O.

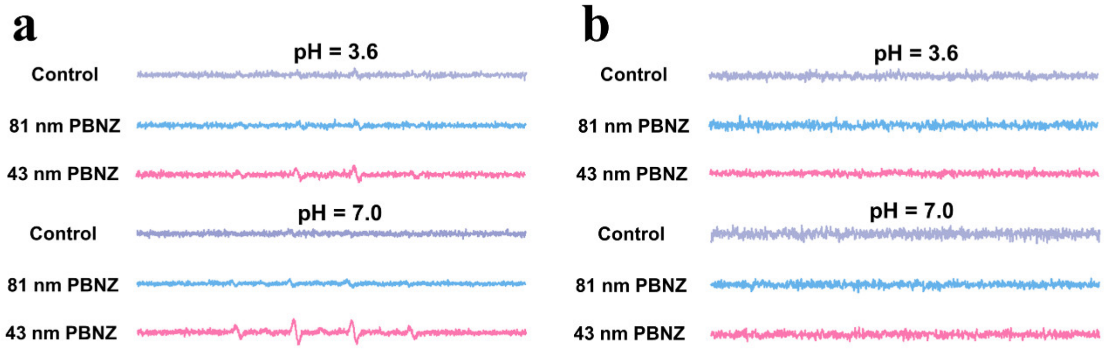

Supplementary Fig. 19 Detection of active intermediates during the interaction of PBNZ with H_2O_2 by EPR. **a** Fe-OH. **b** $^1O_2$.

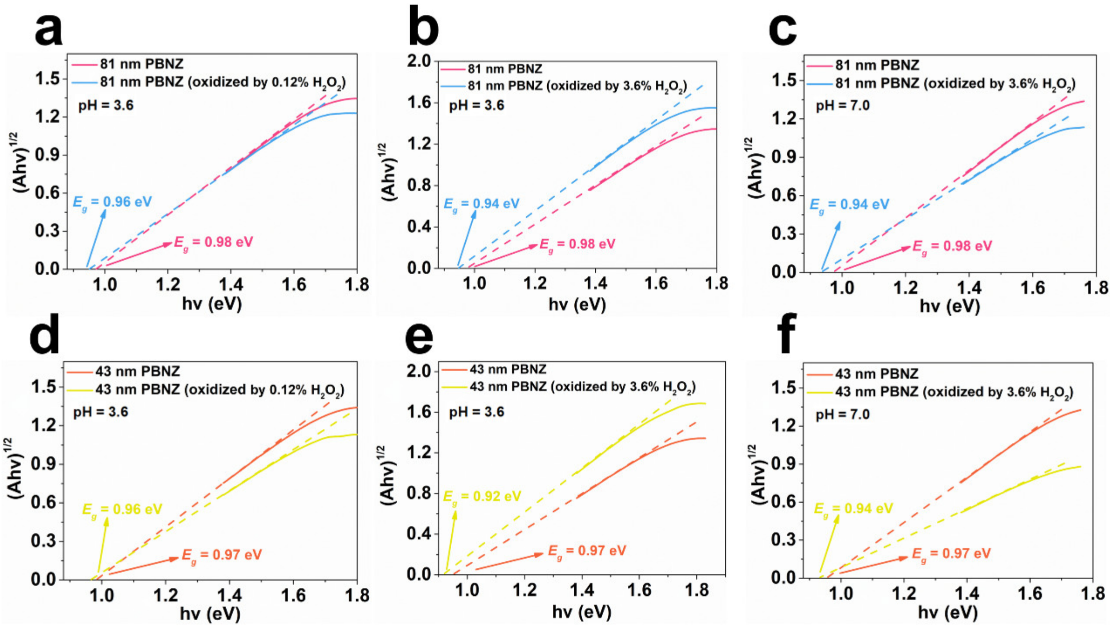

Supplementary Fig. 20 Optical band gap of PBNZ before and after the oxidation of H_2O_2. **a-c** 81 nm PBNZ. **d-f** 43 nm PBNZ.

Supplementary discussion for Supplementary Fig. 20: The E_g decrement after the surface oxidation of PBNZ by 0.12% H_2O_2 was quite weak (Supplementary Fig. 20a, d), which could be suspected as an error of fitting. However, a further decrease was observed when the dosage of H_2O_2 was raised to 3.6% in acidic or neutral environment, confirming the regulating effect of H_2O_2 oxidation on the band gap of PBNZ (Supplementary Fig. 20b, c, e and f). Reported work has compared the E_g value of PB, PY and PW with the relationship as followed: PB < PY << PW[3]. However, the actual existing N-coordinated Fe (II) leads to a partial appearance of PW structures on the surface of PBNZ. Therefore, the minor decrease of E_g could be explained by the oxidation effect of H_2O_2 which transforms the surficial PW to PB. Additionally, the oxidation mainly happened on the surface of particles, while the optical method was used to measure the E_g in the bulk phase of PBNZ. Thus, such bulk phase measurement

would underestimate the variation of the surficial E_g, resulting in the seemingly low decrement of bandgap during the actual detection.

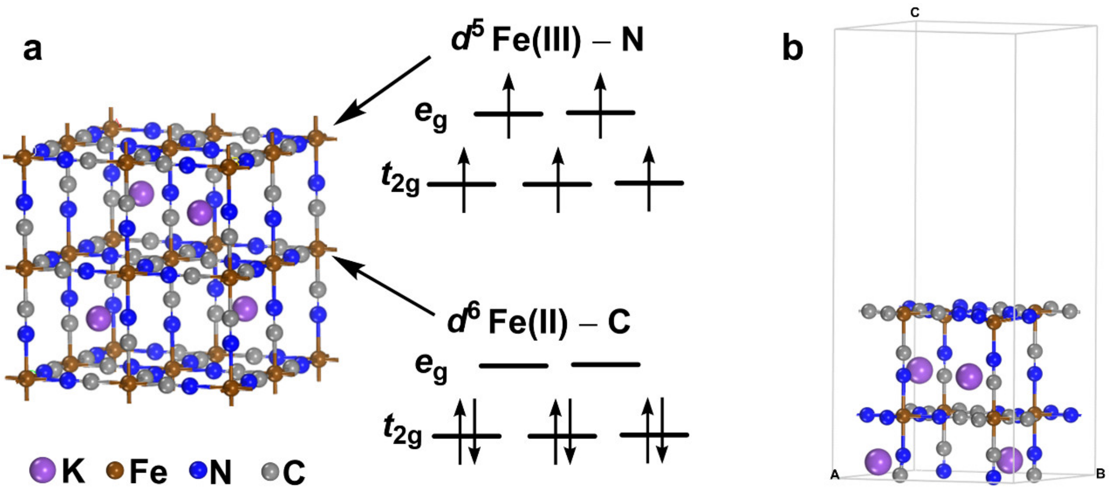

Supplementary Fig. 21 Construction of PBNZ (001) slab model. **a** Bulk structure with electron spin configurations for the N- and C-coordinated Fe atoms. **b** Side view of PBNZ (001) slab.

Supplementary discussion for supplementary Fig. 21: The bulk structure of KFe(III)[(Fe(II)(CN)$_6$], which contained four K-atoms, eight Fe-atoms, and twenty four CN-groups [$K_4Fe_8(CN)_{24}$], was built and geometrically relaxed using the PBE+U method (Supplementary Fig. 21a). The model was similar with previous theoretical studies and real experiment structure[4,5]. The N-coordinated Fe (Fe^{3+}) located at the corners of face-centered cubic (fcc) lattice of the crystal group. The C-coordinated Fe (Fe^{2+}) located at the middle of edge. The difference in the valence state of Fe was compensated by K^+, locating in half of the tetrahedral holes in the lattice. The calculation results showed that the cell parameters of $K_4Fe_8(CN)_{24}$ were: $a = b = c =$ 10.261 Å and $\alpha = \beta = \gamma = 90°$. These values were close to the experimental cell

parameters of PBNZ[4]. The magnetic moments of N- and C-coordinated Fe were 4.467 μ_B and 0.107 μ_B, attributed to the high-spin Fe^{3+} and low-spin Fe^{2+}, respectively. This was consistent with the experimental magnetic data of PBNZ[6].

On the basis of the relaxed bulk structure, the (1×1) PBNZ slab was cut along the (001) direction (Supplementary Fig. 21b). The PBNZ (001) slab also contained four K-atoms, eight Fe-atoms, and twenty four CN-groups [$K_4Fe_8(CN)_{24}$]. The four inner Fe atoms were six-coordinated; the four surface Fe atoms were five-coordinated. The PBNZ (001) slab contained eight atomic layers, and atoms in the bottom four layers were fixed during the subsequent calculations. The magnetic moments of C- and N-coordinated Fe on the surface were 0.052 μ_B and 4.381 μ_B, respectively, which were close to the corresponding magnetic moments of Fe in the lattice. Additionally, H_2O_2 can easily oxidize PB to form ·OH. The ·OH had an adsorption energy of −3.29 eV on surface Fe^{2+} site, whereas CN^- had only an adsorption energy of −1.14 eV. Thus, ·OH also bonded to Fe site on the surface by replacing CN^-. Therefore, the selected PBNZ (001) was a reasonable catalytic reaction model.

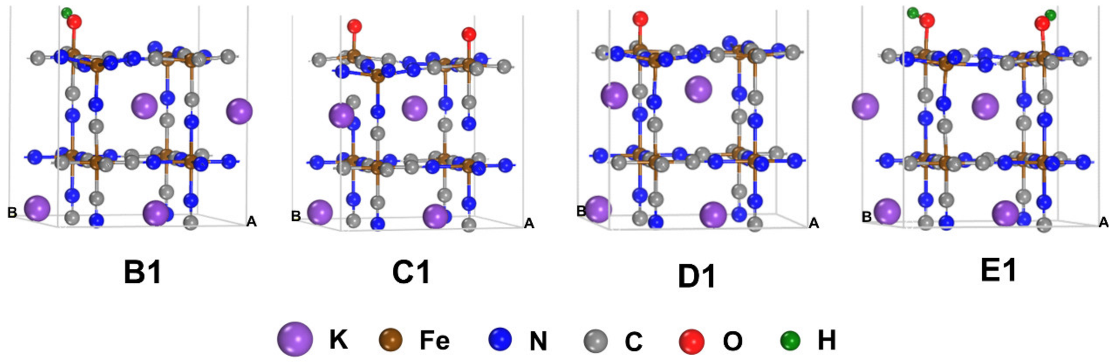

Supplementary Fig. 22 Construction of the PBNZ (001) slab with O or OH group.

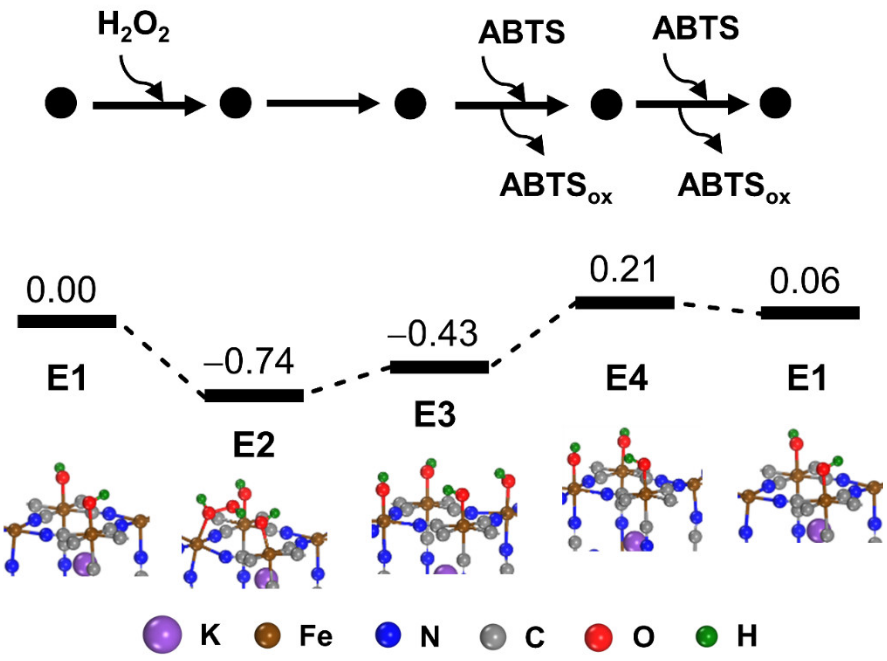

Supplementary Fig. 23 Energy profiles (energy in eV) of VBP initiated by E1.

Supplementary Table 1 Energy levels of PBNZ and catalytic substrates (versus SHE).

No.	Material	E_g (eV)	E_{CB} (eV)	E_{VB} (eV)
1	81 nm PBNZ	0.98	1.22	2.20
2	Oxidized 81 nm PBNZ (0.12% H_2O_2, pH 3.6)	0.96	1.23	2.19
3	Oxidized 81 nm PBNZ (3.6% H_2O_2, pH 3.6)	0.94	1.24	2.18
4	Oxidized 81 nm PBNZ (3.6% H_2O_2, pH 7.0)	0.94	1.24	2.18
5	43 nm PBNZ	0.97	1.23	2.20
6	Oxidized 43 nm PBNZ (0.12% H_2O_2, pH 3.6)	0.96	1.23	2.19
7	Oxidized 43 nm PBNZ (3.6% H_2O_2, pH 3.6)	0.92	1.25	2.17

8	Oxidized 43 nm PBNZ (3.6% H_2O_2, pH 7.0)	0.94	1.24	2.18
			E (eV)	
9	ABTS/$ABTS_{ox}$		0.67[7]	
10	H_2O_2/O_2		0.68	
11	H_2O_2/H_2O		1.77	

Supplementary References

1. Feng, K. et al. Prussian blue nanoparticles having various sizes and crystallinities for multienzyme catalysis and magnetic resonance imaging. *ACS Appl. Nano Mater.* **4**, 5176-5186 (2021).
2. Kang, L. et al. Dual-oxidation-induced lattice disordering in a Prussian blue analog for ultrastable oxygen evolution reaction performance. *J. Colloid Interface Sci.* **630**, 257-265 (2023).
3. Qiu, M. et al. Unveiling the electrochromic mechanism of Prussian Blue by electronic transition analysis. *Nano Energy.* **78**, 105148 (2020).
4. H. J. BUSER, D. SCHWARZENBACH, W. FETTER & LUDI, A. The Crystal Structure of Prussian Blue: $Fe_4[Fe(CN)_6]_3·xH_2O$. *Inorg. Chem.* **16**, 2704−2710 (1977).
5. Hegner, F.S., Galan-Mascaros, J.R. & Lopez, N. A Database of the Structural and Electronic Properties of Prussian Blue, Prussian White, and Berlin Green Compounds through Density Functional Theory. *Inorg. Chem.* **55**, 12851-12862 (2016).
6. F. HERREN, P. FISCHER, A. LUDI & HALG, W. Neutron Diffraction Study of Prussian Blue, $Fe_4[Fe(CN)_6]_3·xH_2O$. Location of Water Molecules and Long-Range Magnetic Order. *Inorg. Chem.* **19**, 956–959 (1980).
7. Bourbonnais, R., Leech, D. & Paice, M.G. Electrochemical analysis of the interactions of laccase mediators with lignin model compounds. *Biochim. Biophys. Acta.* **1379**, 381-390 (1998).
